# Estimating long-term clinical effectiveness and cost-effectiveness of HPV 16/18 vaccine in China

**DOI:** 10.1186/s12885-016-2893-x

**Published:** 2016-11-04

**Authors:** Qian Zhang, Yi-Jun Liu, Shang-Ying Hu, Fang-Hui Zhao

**Affiliations:** 1 Department of Cancer Epidemiology, National Cancer Center/Cancer Hospital, Chinese Academy of Medical Sciences and Peking Union Medical College, Beijing, 100021 China; 2 Department of Preventive Medicine, School of Public Health, Zunyi Medical College, Zunyi, 563099 China

**Keywords:** Cervical cancer, HPV vaccine, Cost-effectiveness

## Abstract

**Background:**

Human papillomavirus (HPV) 16 and 18 are the two most common HPV oncogenic types that can be prevented by vaccination. This study aimed at assessing the cost-effectiveness of 3 doses of the bivalent HPV vaccine in rural and urban settings in China.

**Methods:**

A Markov model was adapted to reflect the lifetime of a modelled 100,000 12-year-old girls cohort in rural and urban settings in China. Input parameters were obtained from published literature, official reports and a two-round expert review panel. Clinical and economic outcomes of vaccination at age 12 with screening was compared to screening only. In the base case analysis, a 3 % discount rate, the vaccine cost of 247 CNY (US$ 39, PAHO vaccine cost in 2013), two rounds of screening in a life time and 70 % coverage for both screening and vaccination were used. One-way, two-way and probabilistic sensitivity analyses were performed. We used different thresholds of cost-effectiveness to reflect the diversity of economic development in China.

**Results:**

Vaccination in addition to screening could prevent 60 % more cervical cancer cases and deaths than screening only. The incremental cost effectiveness ratio varied largely when changing cost of vaccination and discount in one way analysis. Vaccination was very cost-effective when the vaccine cost ranged 87-630 CNY (US$ 13.8-100) in rural and 87-750 CNY (US$ 13.8–119) in urban; and remained cost-effective when the vaccine cost ranged 630–1,700 CNY (US$ 100–270) in rural and 750–1,900 CNY (US$ 119–302) in urban in two way analysis. Probabilistic sensitivity analyses showed that model results were robust.

**Conclusions:**

In both rural and urban, the vaccination cost and discounting are important factors determining the cost-effectiveness of HPV vaccination; policy makers in China should take these into account when making a decision on the introduction of HPV vaccine. In areas with a high burden of cervical cancer and limited screening activities, HPV vaccination should be prioritized. However, the vaccine cost needs to be reduced in order to make it very cost-effective and affordable as well, in particular in poverty areas with high disease burden.

**Electronic supplementary material:**

The online version of this article (doi:10.1186/s12885-016-2893-x) contains supplementary material, which is available to authorized users.

## Background

Given its large population, China accounted for 11.7 % of the world’s cervical cancer (CC) cases and 11.3 % of the world’s CC deaths in 2012 [1]. Since 2009, a screening programme has been implemented in rural China targeting women aged 35 to 64 years, which has gradually expanded from screening 10 million women over a period of 3 years to screening 10 million women per year. Women in urban areas have access to CC screening on an opportunistic basis or through their employment. However, the mortality was reported to increase at an annual rate of 4.1% in young urban women aged 35 to 44 [2]. Furthermore, there are also wide disparities in CC incidence, mortality and screening accessibility between rural and urban areas in China [3].

Persistent HPV infection is the main cause of CC [4]. Seventy percent of all CC worldwide is caused by HPV 16/18 [5]; in China, HPV 16/18 infection accounts for 84.5 % of squamous cell carcinoma [6]. Prophylactic HPV vaccines may add to current efforts of screening in reducing CC burden in China. There are currently two vaccines available globally: a bivalent vaccine produced by GSK (Cervarix^®^) and a quadrivalent vaccine produced by Merck (Gardasil^®^). Both vaccines protect against HPV types 16 and 18 while the quadrivalent vaccine also protects against non-oncogenic types 6 and 11. Cervarix^®^, a bivalent vaccine (GSK, Wavre, Belgium) has been widely used in over 100 countries through regional or national immunization programs to prevent HPV 16/18 infection and related diseases. Cervarix^®^ has shown high immunogenicity and safety, and induces a high degree of protection against HPV-16/18 infection and associated cervical lesions [7, 8]. To date, only Cervarix^®^ has just been approved by Chinese Food and Drug Administration in July 2016 after completation of phase III clinical trials using 3 doses of bivalent HPV vaccination (3DBV) in mainland China [9, 10].

Because the HPV vaccine is relatively costly [11], it has been subject to careful scrutiny. With the increasing importance of economic evaluation in priority setting for health [12], the cost-effectiveness of vaccination strategies should be considered before inclusion in national programmes, as recommended by the World Health Organization (WHO) position paper on HPV vaccines [13]. Mathematical models can help to estimate the long term effectiveness of HPV vaccination in parallel to clinical trials. Such models use a simplified description of the natural history leading to cervical cancer and provide a formal framework to synthesize information from various sources [14]. Modelling studies can integrate currently available clinical data with country-specific epidemiological data to evaluate the potential long-term impact of adding vaccination to screening [15].

Given the important resource implications of introducing the HPV vaccine in China, the financing of the HPV vaccination programme needs to be carefully considered to ensure the best use of resources compared to other priorities in China [16–18]. To date, there have been few comprehensive analyses evaluating the long-term impact of the HPV vaccine in China [19, 20]. Furthermore China is characterized by substantial differences in economic development and disease distribution between regions and therefore the diversity of economic, the ability to pay and screening influence should be considered to achieve the health care fairness. In such context, it is important to advance equity in China’s health system if implementing HPV vaccination in national vaccination program. We aimed to estimate the cost-effectiveness of HPV vaccination using Chinese specific parameters.

## Methods

### Model structure

This study was approved by the Human Subjects Review Boards (Approval No. 13-066/742) of the Cancer Institute and Hospital, Chinese Academy of Medical Sciences (CICAMS). We used a Markov model developed in Microsoft Excel to evaluate the long-term clinical effectiveness and cost-effectiveness of the bivalent vaccine in both rural and urban China for a hypothetical cohort of 100,000 12-year-old girls (Additional file 1: Figure S1).

The Markov model was adapted from previously published models by Debicki [21] and Konno [22] and has been used to perform cost-effectiveness analyses in other countries such as Canada, Taiwan and Japan. The model simulated the natural history of cervical cancer (extending from infection to death) in the hypothetical cohort of 12-year-old girls who are vaccinated in addition to current screening or screening only for cervical cancer, with follow up for a life-time. The Markov model has a cycle time of 1 year and run over life-time of the cohort according to the mortality rate for women reported by National Bureau of Statistics of China [23]. Study parameters were obtained through expert review, literature review and data extraction from previous studies. The study did not collect information from patients and informed consent was therefore not needed. A 3 % discount rate was used with a range (0–5 %) for sensitivity analysis according to the WHO guidelines [24]. The same discount rates for health outcomes and costs were used.

### Perspective

Globally, cervical cancer is considered an important public health problem and disease burden could be reduced by HPV vaccines. In developing countries, the main challenge for the introduction of the HPV vaccine is its price. Evidence from Mexico, Panama and other developing countries showed high vaccination coverage when including HPV vaccine in the national immunization programs by government [25]. Based on the successful experience of hepatitis B vaccine in China, we assumed the government would strengthen the vaccination of HPV according to the China National Plan for NCD Prevention and Treatment [26]. We expect our cost-effectiveness modelling analysis could benefit the health policy, especially public health policy decisions and budget-impact. Based on the considerations above, we chose the health care payer perspective [27] and considered only direct medical costs.

### Model validation

**Fig. 1:**
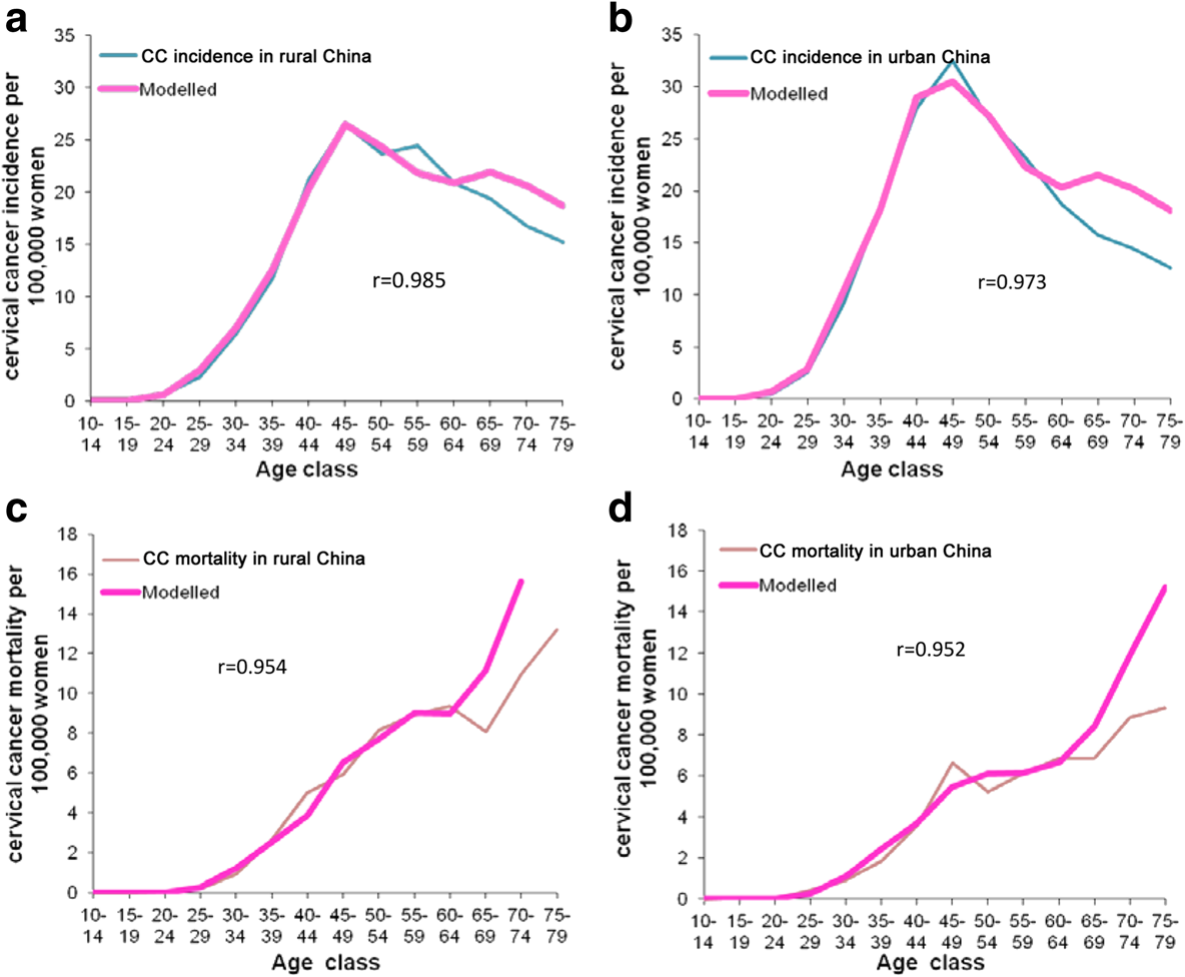
Comparison of data generated from the model with the Chinese cancer registry report (**a**: CC incidence in rural, **b**: CC incidence in urban, **c**: CC mortality in rural, **d**: CC mortality in urban)

The model was validated by comparing the modelled age-specific incidence and mortality of CC in rural and urban China with national register and statistical data [3, 28]. As shown in Fig. 1, the results showed that the modelled cervical cancer incidence and mortality were not significantly different from cancer registry data (*r* = 0.985, 0.973, 0.954 and 0.952 for rural incidence, urban incidence, rural mortality and urban mortality respectively).

### Base case analysis

The base case analysis was conducted for a hypothetical cohort of 100,000 12-year-old girls. We considered two scenarios: HPV vaccination in addition to screening against screening only, separately for rural and urban areas. The cost-effectiveness of HPV vaccination was evaluated by comparing the two scenarios.

The screening and treatment costs were collected by a micro-costing approach [29] from previous studies [30, 31] to estimate aggregated costs associated with cervical cancer, and re-evaluated by a two round expert review panel. Since most patients in China with diagnostically confirmed CC seek treatment in urban hospitals, only the treatment cost of CC in urban areas was estimated. Costs were calculated in Chinese Yuan (CNY), and converted to US dollars (US$) at an exchange rate of 1 US$ = 6.3 CNY for 2013. The two round expert panel lasted for five months. Eight rural and twelve urban clinical gynecologists, epidemiologists and economists chosen from Northern (Beijing, Tianjin, Liaoning), Central (Henan, Shanxi, Jiangsu), Western (Xinjiang) and Southwestern (Sichuan) of China were selected to evaluate the costs of screening, costs of treatment and the proportions of women receiving or refusing the treatment procedures for cancer patients [32].

China is not a member of the Global Alliance for Vaccines and Immunization (GAVI) or the Pan American Health Organization (PAHO) [33, 34], but we assumed China could likely negotiate lower public sector prices [35]. Since China’s GDP per capita is similar to most PAHO countries, such as Peru, Brazil and Chile [34, 36], we assumed in the base case analysis a vaccine cost similar to the PAHO cost (247 CNY (US$ 39) in 2013). In addition, since HPV vaccine price has not been available in China, we obtained the unit cost of vaccine administration from a previous publication on hepatitis B vaccination [37]. We assumed that the HPV vaccine would be delivered through the expanded programme on immunisation (EPI) and would rely on the existing management system (personnel, equipment, cold chain etc.). We therefore only considered the incremental cost of adding the HPV vaccine to the EPI programme, and included the cost of salaries, surveillance, propaganda, training, supervision, transportation, cold chain and other equipments that were related to vaccine delivery. The incremental vaccine administration cost for an additional dose of hepatitis B vaccine was 18 CNY (US$ 3) per child per dose [37] and therefore 54 CNY (US$ 9) for 3-doses of the HPV vaccine. The administration cost was assumed to be the same in rural and urban settings, since our analysis was under the scenario of government supported national vaccination program based on the experience of the hepatitis B vaccine.

The utilities in our model have been used in previously published studies [38–42]. The model assumed no decrements in utility from competing morbidities for non-infected, non-disease-stage subjects. The data of transition probabilities were calculated from the CICAMS pooled database which included more than 30,000 women across China of 17 population-based studies in 9 provinces (Shanxi, Beijing, Henan, Jiangxi, Xinjiang, Shanghai, Jiangsu, Gansu and Guangdong) [43], expert panel and derived from the literature [44–48]. In general, HPV infection increases in young women after sexual debut, but a majority proportion of the HPV infection among young female clears on its own. The HPV incidences were calculated from the CICAMS database [43]. We assumed the age of first sexual debut at 15-year-old in Chinese girls (and therefore assumed no HPV infection in girls under the age of 15 years). The CICAMS database showed that HPV incidence increased from 15-year-old to 25-year-old and decreased from 25-year-old to 40-year-old, then increased again from 40 to 60-year-old [43].

The vaccine efficacy parameters varied with different degrees of cervical intraepithelial neoplasia (CIN1, CIN2 and CIN3). A 93.2 % overall vaccine efficacy against CC irrespective of types, 64.9 % efficacy against CIN2/3 irrespective of types and 50.3 % against CIN1 irrespective of types were assumed in the model [49]. The vaccine coverage was assumed at 70 % both in rural and urban according to the hepatitis B vaccination uptake after 3 years on the market.

The 70 % screening coverage was obtained from cross-sectional studies in China [50, 51]. Screening in rural areas is currently done using Pap smear or visual inspection with acetic acid/Lugol’s iodine (VIA/VILI) [52, 53], while in urban areas Pap smear is the only used primary screening method according to the survey and the expert review panel [32, 54]. The screening sensitivities for CIN1 and CIN2/3 were calculated from the CICAMS database [43]. Both screening scenarios assumed in a lifetime screening at ages 35 and 45, according to cervical cancer screening guidelines for developing countries [55].

The main outcome measure used in the model was the ICER (Incremental Cost-effectiveness Ratio = Incremental Cost per Quality-adjusted Life-years (QALYs)). As there are substantial differences in economic development between rural and urban China, and there is no data on GDPs for rural and urban areas separately, the cost-effectiveness threshold should be considered carefully. We evaluated the cost-effectiveness results against 2 different thresholds in the base case analysis: the intervention was considered very cost-effective if the ICER was less than one time the country’s GDP (i.e., 41,908 CNY, US$ 6,652) and cost-effective if the ICER was less than three times the country’s GDP (i.e., 125,723 CNY, US$ 19,956) [55, 56]. Other measures including the number of cervical cancer cases prevented, deaths avoided, and average life-years and QALY gained for one age cohort were studied over lifetime. In addition, accumulated cost and QALY gained per woman, plus the ICER of 3DBV programme versus screening were calculated. The main input parameters are shown in Additional file 2: Table S1.

### One-way sensitivity analysis

One-way sensitivity analysis was performed to account for methodological, structural and parameter uncertainties and assumptions. The changes of values used were listed in Additional file 2: Table S1. In one-way sensitivity analysis, one parameter would be changed in a certain range while other parameters were kept constant. Most parameters were varied by +/- 20 % from the base case, with the exception of the vaccine cost. The vaccine cost (for 3 doses) was varied from a minimum of 87 CNY (US$ 13.8) which is the GAVI cost to 1,900 CNY (US$ 302) which is the Hong Kong listed private sector cost [33, 34]. Other parameters that were examined in the sensitivity analysis included the discount rate (0/5 %), vaccine efficacy (95 % confidence interval, 95 % CI), screening age (-/+ 5 years) and the vaccination age (12/18 years).

### Two-way sensitivity analysis

Two-way sensitivity analysis was conducted by varying both the vaccine cost from 87 CNY (US$ 13.8) to 1,900 CNY (US$ 302) per course and another parameter, either the discount rate (0/5 %), CC mortality (+/- 20 % from the base case), or screening coverage (30/50 % screening coverage). These three parameters were included in the two-way sensitivity analysis for the following reasons: the discount rate has been shown to be an important parameter that may affect cost-effectiveness results; the CC mortality may vary across different regions of China and the screening coverage can be improved with adequate resources and may therefore change over time. According to base case, which using three times national GDP as the threshold of cost-effectiveness and one time national GDP as the threshold of very cost-effectiveness, the two-way sensitivity analysis would also be conducted in the two thresholds. In addition, taking into account the tremendous varieties of China’s current screening coverage in all regions, screening coverage evaluation were paid specially attention in the analyses. As 70 % screening coverage were extracted from well-designed research studies, we set 70 % as the base case screening coverage but for the current screening status, the screening coverage would be extremely low (6.25 % in rural and 21.5 % in urban). As a result, a lower screening coverage (30 and 50 %) would be analysed in two way price threshold sensitivity analyses and 70 % were assumed to be the idealized screening coverage.

### Probabilistic sensitivity analysis (PSA)

In the PSA, all the main input parameters were changed for each calculation. The combined effect of variations in model inputs were explored via multivariate PSA using @Risk^®^ software (Palisade Corporation) in the Excel model. Distributional functions were assigned to each variable for probabilistic sensitivity analysis to evaluate the robustness of the results.

In PSA, the input distributions were set to be normal distributions (limited between 0 and 1 for transition probabilities) when 95 % CI and Standard Deviations (SD) were available. Otherwise, the uniform distribution with a variation +/- 20 % was used when there were no mean, SD or ranges available (Additional file 2: Table S1). In total, 10,000 samples were generated from the assigned distribution.

## Results

Table 1 showed the base case results for the clinical and economic evaluation. Though cervical cancer can be reduced by effective preventive measures, cervical cancer cases would be missed due to the clinical performances of screening methods. Under the circumstances of the current screening methods in rural and urban, there were 747 cases and 354 deaths occurred in rural, 913 cases and 341 deaths occurred in urban respectively in only screening scenario (Table 1). The combination of vaccination with 3 doses of the bivalent HPV vaccine and screening was estimated to prevent 455 and 557 more cervical cancer cases as well as 215 and 208 more deaths than screening only among 100,000 girls, respectively. This result showed that vaccination in addition to screening could prevent 60 % more cervical cancer cases and deaths than screening only in China.

**Table 1:** Base case cost-effectiveness results without discounting and with discount rate 3 % for both costs and benefits

Rural^a^	Screening only (A)	Screening + vaccination (B)	Difference (B-A)	ICER (CNY per QALY gained)	ICER (US$ per QALY gained)
Undiscounted					
Cost (CNY)	64,386,901	48,641,712	−15,745,190		
Life-years	6,822,557	6,826,325	3768		
CC cases	747	292	−455		
CC deaths	354	139	−215		
QALYs	6,821,273	6,825,826	4553	−3458	−549
Discounted					
Cost (CNY)	19,627,341	29,777,012	10,149,672		
QALYs	2,934,012	2,934,905	893	11,365	1804
Urban^a^	Screening only (A)	Screening + vaccination (B)	Difference (B-A)	ICER (CNY per QALY gained)	ICER (US$ per QALY gained)
Undiscounted					
Cost (CNY)	92,026,104	62,240,211	−29,785,893		
Life-years	7,241,077	7,244,914	3837		
CC cases	913	356	−557		
CC deaths	341	133	−208		
QALYs	7,239,157	7,244,167	5011	−5944	944
Discounted					
Cost (CNY)	28,711,710	34,593,492	5,881,781		
QALYs	3,001,172	3,002,133	961	6124	972

*CC* Cervical Cancer, *QALYs* Quality-adjusted Life-years, *CNY* Chinese Yuan, *US$* United States Dollar, *ICER* Incremental Cost-effectiveness Ratio

^a^100,000 subjects

Under the circumstance that girls were regularly screened twice in their life time, considering the 3 % discount rate, 70 % screening and vaccination coverage and PAHO cost, HPV vaccination had an ICER of 11,365 CNY (US$ 1804) in rural and 6124 CNY (US$ 972) in urban, which is much lower than the national GDP 2013 (41,908 CNY, US$ 6652), which meant that HPV vaccination was very cost effective in this context.

### Sensitivity analyses

**Fig. 2:**
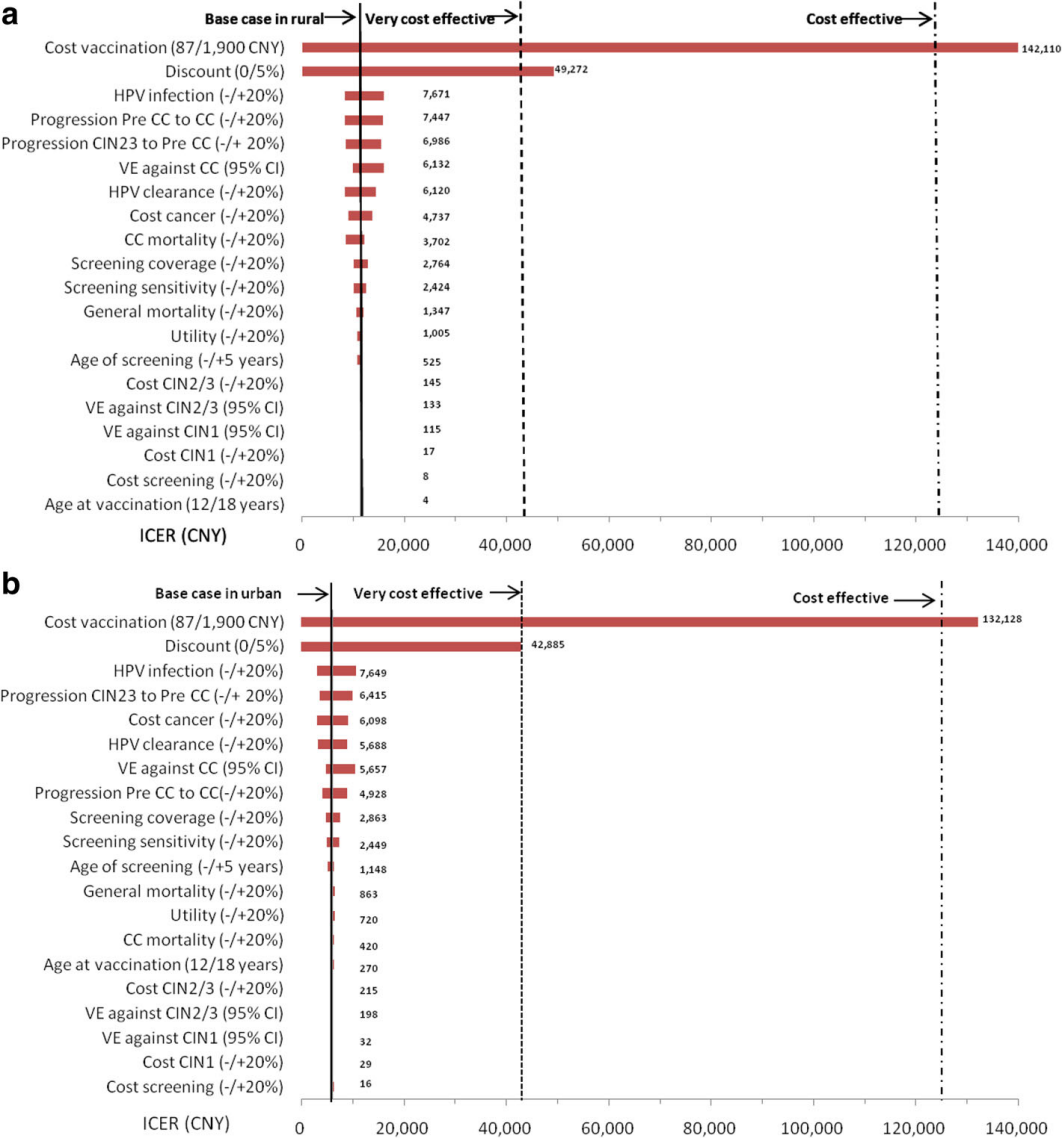
One-way sensitivity analysis: effects of varying factors on ICER in rural settings and urban settings. **a** One-way sensitivity analysis in rural settings. **b** One-way sensitivity analysis in urban settings. CC = cervical cancer; Pre CC to CC = transition probabilities from pre-cancer to cancer; CIN 23 to CC = Progression rate from CIN2/3 to pre-cancer; VE against CC = vaccine efficacy against cervical cancer; VE against CIN1 = vaccine efficacy against CIN1; VE against CIN2/3 = vaccine efficacy against CIN2/3

The most influential factors on whether adding HPV vaccination to screening are cost-effective or not were evaluated in Fig. 2. The ICER varied largely when changing cost of vaccination (ICER ranged 142,110 CNY in rural and 132,128 CNY in urban), and discount (ICER ranged 49,272 CNY in rural and 42,885 CNY in urban) in one way sensitivity analysis. Other parameters, such as CC mortality, HPV infection, progression rate, vaccine efficacy, treatment cost of precancerous lesions, utility, age of screening and age at vaccination had little influence on the cost-effectiveness results. Shown from the one-way sensitivity analysis, when the CC mortality decreased, the HPV infection decreased, the vaccine efficacy against all cervical cancers irrespective of type decreased or the screening sensitivity increased, the vaccine cost should be reduced in order to remain the same ICER. In rural, the results showed that the most influential factors were cost of vaccination, discount rate, HPV infection rate, progression rate and vaccine efficacy (See Panel a). In urban, the most influential factors were cost of vaccination, discount rate, HPV infection rate, progression rate and cost of cancer (See Panel b). For screening coverage additionally, HPV vaccination was still very cost-effective (ICER = 14,761 CNY, US $ 2343 in rural and ICER = 9702 CNY, US$ 1540 in urban) with PAHO cost even in areas with 100 % screening coverage. In areas with the current low coverage (6.25 % [57] in rural and 21.5 % in urban [28]), the ICER would be 6463 CNY (US$ 1026) and 2135 CNY (US$ 339) in rural and urban areas, respectively. Under this circumstance, the 3DBV was estimated to be very cost-effective in both rural and urban.

**Fig. 3:**
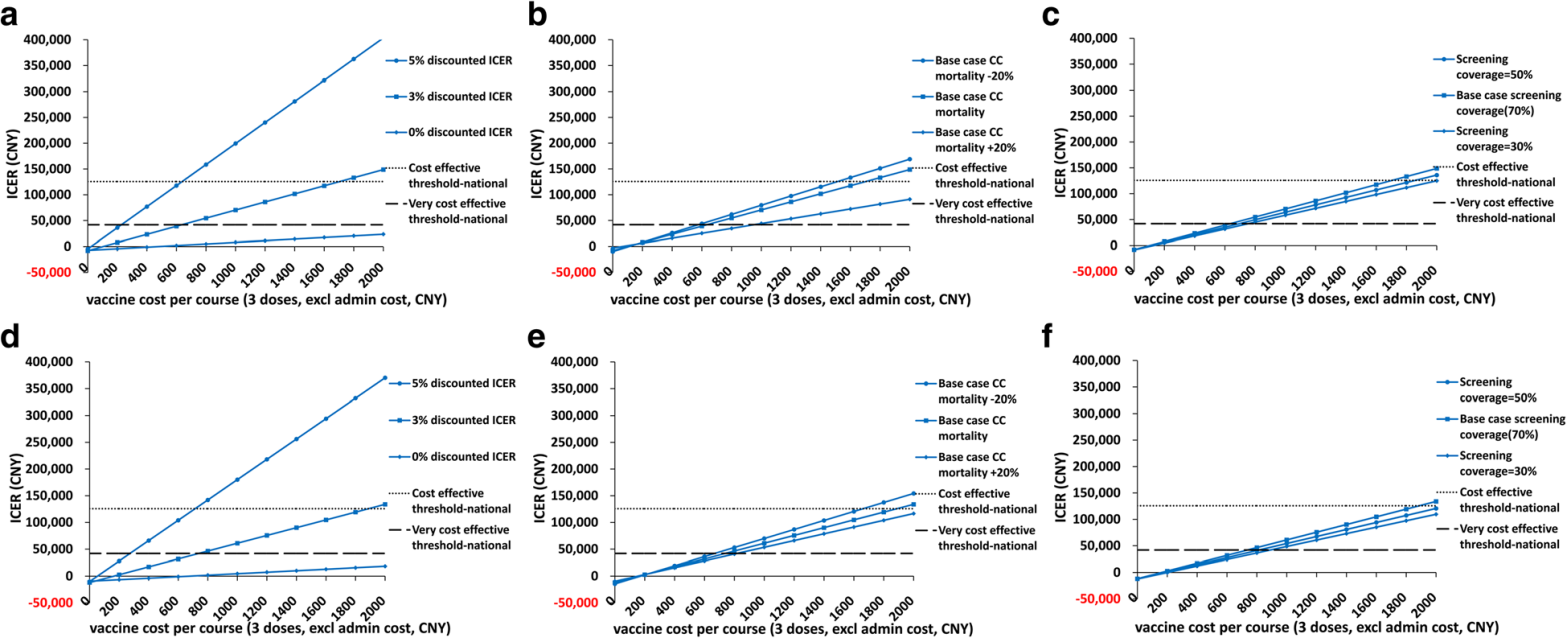
Effects of vaccine price and main factors in rural settings and urban settings. **a** Vaccine cost and discount rate effects in rural. **b** Vaccine cost and CC mortality effects in rural. **c** Vaccine cost and screening coverage effects in rural. **d** Vaccine cost and discount rate effects in urban. **e** Vaccine cost and CC mortality effects in urban. **f** Vaccine cost and screening coverage effects in urban

The two-way sensitivity analysis showed the relationship between vaccine cost and three parameters identified in the one-way sensitivity (Fig. 3). Taken rural areas (Panel a, b, c) as an example, in panel a, the blue line with round dot was the results with 5 % discount; the blue line with square dot was the results with 3 % discount (base case value); the blue line with the diamond dot was the results with 0 % discount. The black lines showed the thresholds of cost effectiveness (three times national GDP) and very cost effectiveness (one time national GDP). Shown from panel a with 3 % discount, HPV vaccination was very cost-effective if the price was lower than 630 CNY (US$ 100) and it was cost-effective if the price was lower than 1700 CNY (US$ 270) in rural areas. With discount rate increased, the cost of HPV vaccine should be reduced to be cost-effective. On the other hand, with discount rate decreased, it was still cost-effective with the price of 1900 CNY (US$ 302). Similarly, for areas with higher CC mortality, HPV vaccination could be more cost-effective than in areas with lower CC mortality (See panel b). For areas with extremely lower screening coverage, HPV vaccination could be more cost-effective than in areas with higher screening coverage (See panel c). In urban, vaccination varied from very cost-effective to cost-effective with the national threshold when the vaccine cost ranged from 750 CNY (US$ 119) to 1900 CNY (US$ 302) in base case (Fig. 3, panel d, e, f).

**Fig. 4:**
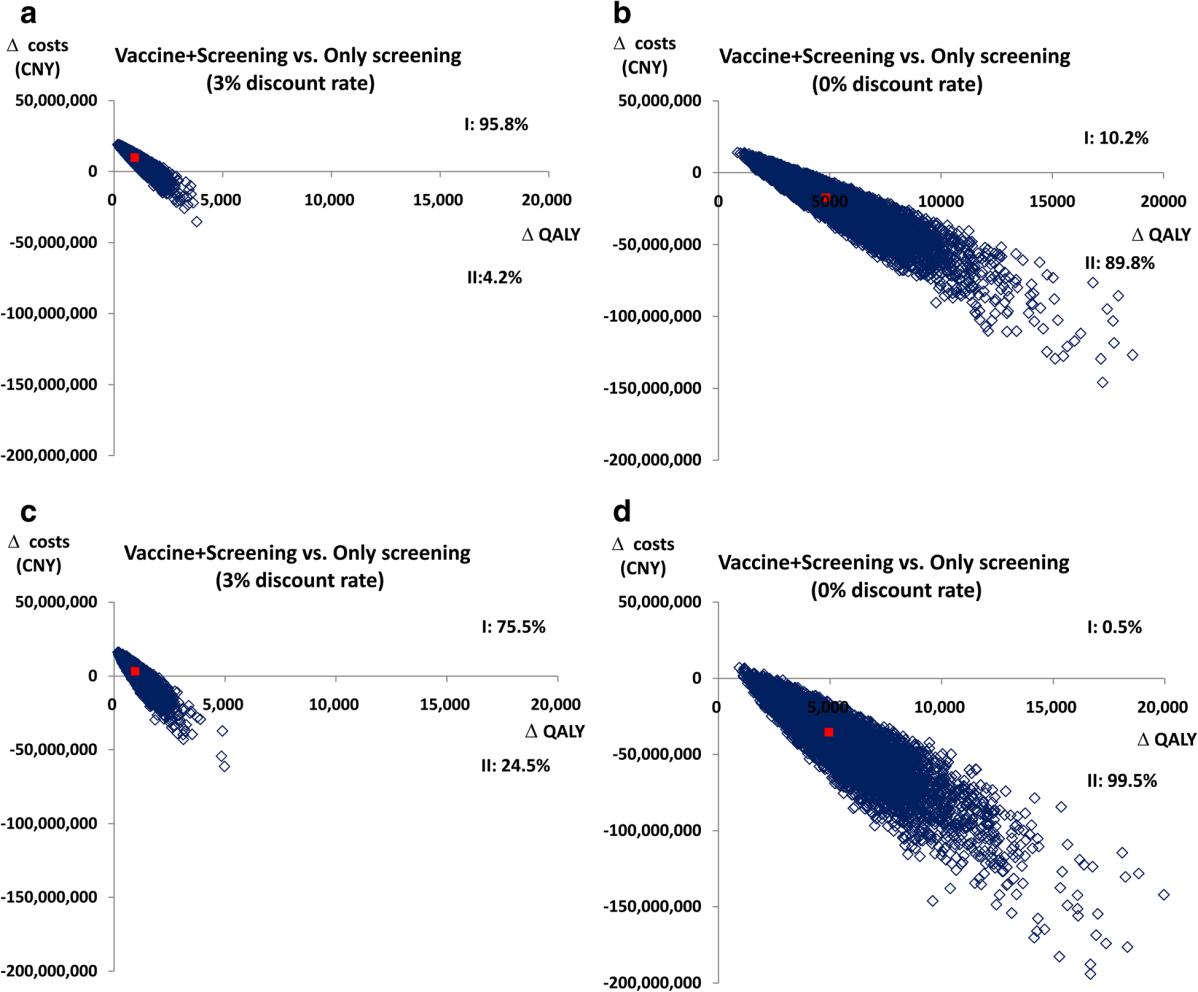
Probabilistic sensitivity analyses in rural and urban. **a** Probabilistic sensitivity analyses with discounted in rural. **b** Probabilistic sensitivity analyses with undiscounted in rural. **c** Probabilistic sensitivity analyses with discounted in urban. **d** Probabilistic sensitivity analyses with undiscounted in urban. (Note: X-axis represents the incremental QALYs and y-axis represents the incremental costs. Each blue dot on the graph represents the relative discounted/undiscounted ICER of one out of the 10,000 simulations and the red dot represents the average value. Quadrant I represents comparing with screening only, vaccine and screening would gain more QALYs with the costs increasing. Quadrant II represents comparing with screening only, vaccine and screening would gain more QALYs with the costs decreasing. Quadrant III represents comparing with screening only, vaccine and screening would lose more QALYs with the costs decreasing. Quadrant IV represents comparing with screening only, vaccine and screening would lose more QALYs with the costs increasing. Quadrant II means vaccine and screening would be more cost-effective compared with screening only. Quadrant IV means screening only would be more cost-effective compared with vaccine and screening)

Figure 4 showed the PSA in rural and urban. Each blue dot on the graph represented the relative discounted/undiscounted ICER of one out of the 10,000 simulations. X-axis represented the incremental QALYs and y-axis represented the incremental costs. The red dots represented the average value and the blue dots represented each replicates.

For rural setting, when comparing vaccination in addition to screening with screening only, with 3 % discount, 95.8 % of the replicates predicted to have greater lifetime costs and greater QALY benefit than the screening only scenario (Quadrant I of Fig. 4a). 4.2 % of dots fell into Quadrant II meant that vaccine combined with screening would cost less but gained more QALYs than screening only (Quadrant II in Fig. 4a). When undiscounted, 10.2 % of the replicates predicted to have greater lifetime costs and greater QALY benefit than the screening only in rural (Quadrant I of Fig. 4b). 89.8 % of dots fell into Quadrant II meant that vaccine combined with screening would cost less but gained more QALYs than screening only (Quadrant II in Fig. 4b). For urban setting, when comparing vaccination in addition to screening with screening only, with 3 % discount, 75.5 % of the replicates predicted to have greater lifetime costs and greater QALY benefit than the screening only scenario (Quadrant I of Fig. 4c). 24.5 % of dots fell into Quadrant II meant that vaccine combined with screening would cost less but gained more QALYs than screening only (Quadrant II in Fig. 4c). When undiscounted, 0.5 % of the replicates predicted to have greater lifetime costs and greater QALY benefit than the screening only in urban (Quadrant I of Fig. 4d). 99.5 % of dots fell into Quadrant II meant that vaccine combined with screening would cost less but gained more QALYs than screening only (Quadrant II in Fig. 4d).

The results showed that most of the replicates were in Quadrant I with 3 % discount and Quadrant II with 0 % discount, showing that vaccination in addition to screening had greater QALY benefit compared to screening only. All the replicates were less than the threshold of 3 times national GDP, that represented 100 % of replicates were cost-effectiveness in rural and urban respectively.

## Discussion

The ongoing screening programme with conventional Pap smear or visual inspection will inevitably miss many pre-cancer lesions due to the limitations of these two methods and the lack of health care workers [58]. Moreover, almost ten years delay of the HPV vaccine in China would cause more than 59 million Chinese girls aged 9-15 to lose their opportunities for vaccination [35] and put 206,000 girls at high risk for cervical cancer over the next 25 years [59]. HPV vaccine may add to current efforts of screening in reducing cervical cancer burden in China in the future.

In the current screening programme, considering the different situation in rural and urban areas, our study analysed two screening strategies respectively to reflect the difference in screening practice between rural and urban settings. Input parameters for rural and urban differed by HPV incidence, proportion of precancers treated, screening and treatment costs for precancerous lesions. The vaccine coverage was assumed the same (70 %) in rural and urban based on the perspective that government would include HPV vaccine in the national vaccination program as well as the success experiences of Hepatitis B vaccine in China. We also set the same methods for cancer treatment between rural and urban, given the realistic situation that most of the rural patients with diagnostically confirmed cervical cancer would seek for treatment in the urban hospitals due to no cancer therapeutic capacity in rural areas. The administration cost was US$ 9 per course, which was similar to previous published studies [18, 60]. The main findings showed that vaccination with 70 % coverage of 3DBV in 12-year-old girls in addition to screening could prevent 60 % more cervical cancer cases and deaths than screening only in China. In a case-control study in United States [61], the prevalence of HPV16/18 in CIN2+ lesions was demonstrated to decrease from 53.6 to 28.4 % among women received at least one dose of HPV vaccine (*P*_*trend*_ < 0.001), which could lead to the reduction of cervical cancer in the future.

The perspective of health care payer was used in this study. Compared with societal perspective which considered the patient time, travel costs, willingness-to-pay and many other indirect parameters, the health care payer perspective could provide more information for the informing budget impact and policy decisions. As decisions on introduction of vaccines are often influenced by funding agencies, analysis such as budget impact assessments focusing on cost-saving, affordability and sustainability are relevant in resource-constrained settings [18, 62, 63]. The costs borne by providers (e.g., donors and governments) which separated by patients and their families could allow judgments to be made from the viewpoints of the various decision-makers. This is particularly important for middle and low income countries that may be required to fully-finance the cost of vaccines. Lieu [64] estimated (based on existing knowledge about the vaccine at the time) that from the health care payer perspective pneumococcal vaccination of healthy infants in the United States would result in savings if the vaccine cost US$ 18 or less per dose, but from the societal perspective, the vaccination programme would result in savings if the vaccine cost US$ 46 or less per dose. Analysts should therefore be cognizant that whilst a broader perspective that includes productivity losses (gains) will improve cost-effectiveness, it can also be used to justify higher vaccine cost, as it increases the break-even price per dose, i.e., the price at which the cost of the vaccination programme is exactly off-set by the savings due to vaccination [65].

In the sensitivity analysis, whether the results were cost-effective or not depended on the variability of the key input parameters. HPV vaccination was very cost-effective if the price was lower than 630 CNY (US$ 100) in rural and 750 CNY (US$ 119) in urban in two-way analysis. In order to be very cost-effective, the vaccine cost should be reduced in areas with lower cervical cancer mortality and higher screening coverage. Health care payers should compare the economic development level with the national threshold, CC mortality, HPV infection, HPV genotypes distribution, screening coverage, screening methods and other dominant parameters to decide the appropriate vaccination price. Global cost-effectiveness analysis informed by country-based evidence suggests that vaccinating pre-adolescent girls can be cost-effective, particularly in resource-constrained settings where alternative cervical cancer prevention and control measures often have limited coverage [66–69]. In the base case, we assumed the screening coverage as 70 % according to the previous research studies in China [50, 51], the coverage rate in real world setting would be much lower, for an instance, it was only 21.5 % in urban [28] and 6.25 % [57] in rural so far. For areas with low screening coverage and high cervical cancer burden, HPV vaccination could be more cost-effective and more urgent to be included into the vaccination plans.

Due to the diverse economic development of China, the different areas have different affordability profile. Since 2008, Panama has been the first country in Latin America and the Caribbean to provide the HPV vaccine free of charge to young adolescent girls [70]. After that, many developing countries have implemented the HPV vaccine [71, 72] into national programmes. In China, the government would strengthen the vaccination of HPV according to the China National Plan for NCD Prevention and Treatment [26]. However, the vaccine coverage and price would be the critical factors to be considered in the planning of vaccination programme.

In order to promote the implementation of HPV vaccine in China, multiple surveys have been done to gauge HPV vaccine awareness and knowledge. These multi-centre surveys in China showed that there was a high acceptability of HPV vaccine to prevent CC among Chinese women, but the price paid by individual should be considered carefully when the vaccines become available [73, 74]. The vaccine safety and efficacy would be the main concerns of HPV vaccine among Chinese [75]. Further proof of vaccine safety and efficacy and government subsidies combined with increased awareness could facilitate development and implementation of HPV vaccination in China.

Our analyses assumed the cost of 3 doses of HPV vaccine considering the registration clinical trial in China is for 3-dose vaccine regime. While the WHO position paper [13] on HPV vaccines proposed that the adoption of a 2-dose vaccine schedule could be recommended for girls aged 9–13 years. A 2-dose vaccine schedule might be more cost-effectiveness due to the non-inferior efficacy and less cost compared to 3-dose schedule.

Not only one-way sensitivity analysis, but also two-way analysis and probabilistic analysis were performed to certify the stability of the model. The main findings in our study were consistent with the previous published studies that evaluate the cost-effectiveness of HPV vaccination in China [19, 20]. We all found that combined HPV vaccination with screening would be more cost-effectiveness than only screening under a certain vaccine cost. The results in our study showed that adding vaccination to screening would reduce 60 % more cancer, while the other study showed a 44 % cancer reduction [20]. And the discrepancies between our study and others mainly occurred in the input parameters. The parameters in our Markov model were collected by multiple channels and validated by the expert panel, in which the experts came from different regions to fully capture the heterogeneity in disease burden, health systems and socio-economic development of China. The parameters were reliable and stable, which were estimated based on the pooled Chinese databases from multi-centre studies in rural and urban areas of 9 provinces across China [43]. Moreover, we used the most recently updated data from National Bureau of Statistics [23], China Cancer Registry [3] and Statistics of National Health and Family Planning Commission [58].

Limitations also exist in the methods. Firstly, the model did not capture the indirect protection resulting from the herd immunity caused by the reduction of circulation of the infective agent [76]. As a result, the benefits of the vaccination could be underestimated. Secondly, the vaccine efficacy in our model was assumed to be life-long, however,but the vaccine efficacy clinical trials have only lasted less than 10 years, so the duration of vaccine efficacy is unknown. Thirdly, the end study analysis of clinical registration trials of HPV vaccines in China are ongoing [9, 10] and no final data for the vaccine efficacy against CIN2/3+ irrespective of HPV types are available, so we adopted 93 % vaccine efficacy from PATRICIA trial [49]. The assumption of lifelong duration of vaccination and the high efficacy could overestimate the benefits of vaccination and result in more favourable ICERs. Fourthly, one of the uncertainties we have is whether HPV type replacement take place once vaccination against HPV-16/18 is widespread. The prevalence of HPV-16/18 falls to very low levels with vaccination. Other oncogenic HPV subtypes currently responsible for relatively few CC cases, might fill the niche left by HPV-16/18. To date this question could not be answered but available evidence suggests that niche competition is unlikely to happen [77]. Lastly, the present model only included cervical cancer and pre-cancer, irrespective of the vulvar cancer, vaginal cancer, anal and some proportion of oropharyngeal cancer cancers that the HPV vaccine may have efficacy in preventing [78–80]. If these diseases were included in the evaluation, the protection offered by HPV vaccination would be wider, and would lead to a lower ICER than the present analyses. These results are thus likely to provide a conservative estimate.

## Conclusions

In both rural and urban areas, the vaccination cost and discounting are important factors determining the cost-effectiveness of HPV vaccination; policy makers in China should take these into account when making a decision on the introduction of HPV vaccine. In areas with a high burden of cervical cancer and limited screening activities, HPV vaccination should be prioritized. However, the vaccine cost needs to be reduced in order to make it very cost-effective and affordable as well, in particular in poverty areas with high disease burden.

## Additional files

Additional file 1: Figure S1. Lifetime cohort Markov model adapted to the rural and urban settings in China. Note: CIN: cervical intraepithelial neoplasia; CIN1onc: cervical intraepithelial neoplasia 1; HPV: human papillomavirus; HPVonc: oncogenic HPV infection; NoHPVonc: no oncogenic HPV infection. (JPG 24 kb)
Additional file 2: Table S1. Input data values for base case, one-way sensitivity analyses and probabilistic sensitivity analyses. ^a^ Screening practice: all women screened twice lifetime at 35 years and 45 years. Health states No HPV, HPV, CIN 1 and CIN 2/3 have utility=1 (i.e., no disutility); health states death and death from cervical cancer have utility=0; PSA=probabilistic sensitivity analyses; CIN=cervical intraepithelial neoplasia; HPV=human papillomavirus; CC=cervical cancer; VIA/VILI=visual inspection with acetic acid/iodine; NA=Not include in probabilistic sensitivity analyses; Se=sensitivity. (DOCX 21 kb)

## Abbreviations

3DBV: 3 Doses of Bivalent HPV Vaccination
CC: Cervical Cancer
CI: Confidence Interval
CICAMS: Cancer Institute and Hospital, Chinese Academy of Medical Sciences
CIN: Cervical Intraepithelial Neoplasia
CNY: Chinese Yuan
EPI: Expanded Programme on Immunisation
GAVI: Global Alliance for Vaccines and Immunization
GDP: Gross Domestic Product
HPV: Human Papillomavirus
ICER: Incremental Cost-effectiveness Ratio
NA: Not included in probabilistic sensitivity analyses
PAHO: Pan American Health Organization
PATRICIA: PApilloma TRIal against Cancer In young Adults
PSA: Probabilistic Sensitivity Analyses
QALYs: Quality-adjusted Life-years
SD: Standard Deviations
US$: United States Dollar
VIA/VILI: Visual Inspection with Acetic Acid/Lugol’s Iodine
WHO: World Health Organization

## References

[1] Ferlay J, Soerjomataram I, Ervik M, Dikshit R, Eser S, Mathers C, et al. GLOBOCAN 2012 v1.0, Cancer Incidence and Mortality Worldwide: IARC Cancer Base No. 11 [Internet]. Lyon, France: International Agency for Research on Cancer; 2013. http://globocan.iarc.fr. Accessed 26 Dec 2014.

[2] Yang L, Parkin DM, Li L, Chen Y (2003). Time trends in cancer mortality in China: 1987-1999. Int J Cancer.

[3] Chen WQ, Zheng RS, Zeng HM, Zou XN, Zhang SW, He J (2015). [Report of cancer incidence and mortality in china, 2011] [in Chinese]. China Cancer.

[4] Zur Hausen H (1987). Papillomaviruses in human cancer. Appl Pathol.

[5] Muñoz N, Bosch FX, de Sanjosé S, Herrero R, Castellsagué X, Shah KV (2003). Epidemiologic classification of human papillomavirus types associated with cervical cancer. N Engl J Med.

[6] Chen W, Zhang X, Molijn A, Jenkins D, Shi JF, Quint W (2009). Human papillomavirus type-distribution in cervical cancer in China: the importance of HPV 16 and 18. Cancer Causes Control.

[7] Paavonen J, Naud P, Salmerón J, Wheeler CM, Chow SN, Apter D (2009). Efficacy of human papillomavirus (HPV)-16/18 AS04-adjuvanted vaccine against cervical infection and precancer caused by oncogenic HPV types (PATRICIA): final analysis of a double-blind, randomised study in young women. Lancet.

[8] Harper DM, Franco EL, Wheeler CM, Moscicki AB, Romanowski B, Roteli-Martins CM (2006). Sustained efficacy up to 4.5 years of a bivalent L1 virus-like particle vaccine against human papillomavirus types 16 and 18: follow-up from a randomised control trial. Lancet.

[9] Zhu FC, Chen W, Hu YM, Hong Y, Li J, Zhang X (2014). Efficacy, immunogenicity and safety of the HPV-16/18 AS04-adjuvanted vaccine in healthy Chinese women aged 18-25 years: results from a randomized controlled trial. Int J Cancer.

[10] ClinicalTrials.gov. http://www.clinicaltrials.gov. Accessed 21 Jan 2015.

[11] Arie S (2011). Global HPV, vaccination. BMJ.

[12] Jit M, Brisson M (2011). Modelling the epidemiology of infectious diseases for decision analysis: a primer. Pharmacoeconomics.

[13] WHO position paper. http://www.who.int/immunization/documents/positionpapers/en/. Accessed 11 Jan 2016.

[14] Brisson M, Edmunds WJ (2006). Impact of model, methodological, and parameter uncertainty in the economic analysis of vaccination programs. Med Decis Making.

[15] Demarteau N, Detournay B, Tehard B, El Hasnaoui A, Standaert B (2011). A generally applicable cost-effectiveness model for the evaluation of vaccines against cervical cancer. Int J Public Health.

[16] Jit M, Demarteau N, Elbasha E, Ginsberg G, Kim J, Praditsitthikorn N (2011). Human papillomavirus vaccine introduction in low-income and middle-income countries: guidance on the use of cost-effectiveness models. BMC Med.

[17] Goldie SJ, O’Shea M, Campos NG, Diaz M, Sweet S, Kim SY (2008). Health and economic outcomes of HPV 16,18 vaccination in 72 GAVI-eligible countries. Vaccine.

[18] Jit M, Levin C, Brisson M, Levin A, Resch S, Berkhof J (2013). Economic analyses to support decisions about HPV vaccination in low- and middle-income countries: a consensus report and guide for analysis. BMC Med.

[19] Canfell K, Shi JF, Lew JB, Walker R, Zhao FH, Simonella L (2011). Prevention of cervical cancer in rural China: evaluation of HPV vaccination and primary HPV screening strategies. Vaccine.

[20] Levin CE, Sharma M, Olson Z, Verguet S, Shi JF, Wang SM (2015). An extended cost-effectiveness analysis of publicly financed HPV vaccination to prevent cervical cancer in China. Vaccine.

[21] Debicki D, Ferko N, Demarteau N, Gallivan S, Bauch C, Anonychuk A (2008). Comparison of detailed and succinct cohort modelling approaches in a multi-regional evaluation of cervical cancer vaccination. Vaccine.

[22] Konno R, Sasagawa T, Fukuda T, Van Kriekinge G, Demarteau N (2010). Cost-effectiveness analysis of prophylactic cervical cancer vaccination in Japanese women. Int J Gynecol Cancer.

[23] National Bureau of Statistics of the People’s Republic of China. http://www.stats.gov.cn/tjsj/pcsj/rkpc/6rp/indexch.htm. Accessed 15 May 2013.

[24] Jeffrey DS (2001). Macroeconomics and health. Health: investing in health for economic development.

[25] Muñoz N. Progress in HPV vaccine introduction in Latin America. HPV TODAY. 2012. http://www.hpvtoday.com. Accessed 10 Dec 2014.

[26] China National Plan for NCD Prevention and Treatment (2012-2015). http://www.chinacdc.cn/en/ne/201207/t20120725_64430.html. Accessed 12 Dec 2014.

[27] China Guidelines for Pharmacoeconomic Evaluations. http://www.ispor.org/PEguidelines/source/China-Guidelines-for-Pharmacoeconomic-Evaluations_2011_Chinese.pdf. Accessed 20 May 2016.

[28] National Bureau of Statistics of China. http://data.stats.gov.cn/easyquery.htm?cn=C01. Accessed 5 Jul 2014.

[29] Shi JF, Chen JF, Canfell K, Feng XX, Ma JF, Zhang YZ (2012). Estimation of the costs of cervical cancer screening, diagnosis and treatment in rural Shanxi Province, China: a micro-costing study. BMC Health Serv Res.

[30] Zhao FH, Chen JF, Gao XH, Gao LM, Liu QG, Liu ZH (2012). [Effectiveness and health economic analysis of strategies on cervical cancer screening and early diagnosis and treatment] [in Chinsese]. Zhonghua Zhong Liu Za Zhi.

[31] Shi JF, Canfell K, Lew JB, Zhao FH, Legood R, Ning Y (2011). Evaluation of primary HPV-DNA testing in relation to visual inspection methods for cervical cancer screening in rural China: an epidemiologic and cost-effectiveness modelling study. BMC Cancer.

[32] Liu YJ, Zhang Q, Hu SY, Zhao FH. Costs of detection and treatment of cervical cancer, cervical intraepithelial neoplasias in China. Asia-Oceania Research Organisation in Genital Infection and Neoplasia (AOGIN) 2014, Beijing, China.

[33] GAVI (Global Alliance for Vaccines and Immunisations). Human papillomavirus vaccine support; http://www.gavi.org/support/nvs/human-papillomavirus/. Accessed 20 Jun 2016.

[34] PAHO (Pan American Health Organization). Expanded Program of Immunization Vaccine Prices for Year 2013- Amendment III.

[35] Colombara DV, Wang SM. The impact of HPV vaccination delays in China: Lessons from HBV control programs. Vaccine 2013;31(38):4057–9. doi: 10.1016/j.vaccine.2013.06.031. 10.1016/j.vaccine.2013.06.031 PMC4015133 23777955

[36] World Economic Outlook Database-April 2016, International Monetary Fund. http://www.imf.org/en/data. Accessed 12 Apr 2016.

[37] Yu WZ, Yu JJ, Cui G, Jin SG, Wag J, Tao Z (2006). [Study on the reasonable cost of national immunization program in some regions of china] [in Chinese]. Chinese JVaccines Immun.

[38] Suárez E, Smith JS, Bosch FX, Nieminen P, Chen CJ, Torvinen S (2008). Cost-effectiveness of vaccination against cervical cancer: a multi-regional analysis assessing the impact of vaccine characteristics and alternative vaccination scenarios. Vaccine.

[39] Goldie SJ, Kohli M, Grima D, Weinstein MC, Wright TC, Bosch FX (2004). Projected clinical benefits and cost-effectiveness of a human papillomavirus 16/18 vaccine. J Natl Cancer Inst.

[40] Gold MR, Franks P, McCoy KI, Fryback DG (1998). Toward consistency in cost-utility analyses: using national measures to create condition-specific values. Med Care.

[41] Trogdon JG, Ekwueme DU, Subramanian S, Crouse W (2013). Economies of scale in federally-funded state-organized public health programs: results from the National Breast and Cervical Cancer Early Detection Programs. Health Care Manag Sci.

[42] Vale DB, Morais SS, Pimenta AL, Zeferino LC (2010). [Assessment of the cervical cancer screening in the Family Health Strategy in Amparo, Sao Paulo State, Brazil. Cad Saude Publica.

[43] Zhao FH, Lin MJ, Chen F, Hu SY, Zhang R, Belinson JL (2010). Performance of high-risk human papillomavirus DNA testing as a primary screen for cervical cancer: a pooled analysis of individual patient data from 17 population-based studies from China. Lancet Oncol.

[44] Moscicki AB, Hills N, Shiboski S, Powell K, Jay N, Hanson E (2001). Risks for incident human papillomavirus infection and low-grade squamous intraepithelial lesion development in young females. JAMA.

[45] Van de Velde N (2007). Brisson M, Boily MC, Modeling human papillomavirus vaccine effectiveness: quantifying the impact of parameter uncertainty. Am J Epidemiol.

[46] Sanders GD, Taira AV (2003). Cost-effectiveness of a potential vaccine for human papillomavirus. Emerg Infect Dis.

[47] Melnikow J, Nuovo J, Willan AR, Chan BK, Howell LP (1998). Natural history of cervical squamous intraepithelial lesions: a meta-analysis. Obstet Gynecol.

[48] Quinn MA, Benedet JL, Odicino F, Maisonneuve P, Beller U, Creasman WT (2006). Carcinoma of the cervix uteri. Int J Gynaecol Obstet.

[49] Lehtinen M, Paavonen J, Wheeler CM, Jaisamrarn U, Garland SM, Castellsagué X (2012). Overall efficacy of HPV-16/18 AS04-adjuvanted vaccine against grade 3 or greater cervical intraepithelial neoplasia: 4-year end-of-study analysis of the randomised, double-blind PATRICIA trial. Lancet Oncol.

[50] Cagle AJ, Hu SY, Sellors JW, Bao YP, Lim JM, Li SM (2010). Use of an expanded gold standard to estimate the accuracy of colposcopy and visual inspection with acetic acid. Int J Cancer.

[51] Qiao YL, Sellors JW, Eder PS, Bao YP, Lim JM, Zhao FH (2008). A new HPV-DNA test for cervical-cancer screening in developing regions: a cross-sectional study of clinical accuracy in rural China. Lancet Oncol.

[52] Cuzick J, Clavel C, Petry KU, Meijer CJ, Hoyer H, Ratnam S (2006). Overview of the European and North American studies on HPV testing in primary cervical cancer screening. Int J Cancer.

[53] Xu H, Zhao FH, Gao XH, Hu SY, Chen JF, Liu ZH (2013). [Cost-effectiveness analysis on the once in a life time cervical cancer screening program for women living in rural and urban areas of China] [in Chinese]. Zhonghua liu xing bing xue za zhi.

[54] Li Q, Liu ZH, Liu ST, Li XL, Huang ME, Ye ML (2008). [Opportunistic Screening for cervical cancer in City Hospital] [in Chinese]. Chinese Cancer.

[55] Cancer, I.A.f.R.o (2005). IARC handbooks of cancer prevention Vol. 10. Cervix cancer screening.

[56] World Health Organization (2008). WHO guide for standardization of economic evaluations of immunization programmes.

[57] Women’s health in rural China. Lancet. 2009;374:358. 10.1016/S0140-6736(09)61394-5 19647592

[58] National Health and Family Planning Commission of the People’s Republic of China. http://www.nhfpc.gov.cn/zwgkzt/pwstj/list.shtml Accessed 15 Oct 2014.

[59] Shi JF, Canfell K, Lew JB, Qiao YL (2012). The burden of cervical cancer in China: synthesis of the evidence. Int J Cancer.

[60] Ogembo JG, Manga S, Nulah K, Foglabenchi LH, Perlman S, Wamai RG (2014). Achieving high uptake of human papillomavirus vaccine in Cameroon: lessons learned in overcoming challenges. Vaccine.

[61] Hariri S, Bennett NM, Niccolai LM, Schafer S, Park IU, Bloch KC (2015). Reduction in HPV 16/18-associated high grade cervical lesions following HPV vaccine introduction in the United States - 2008-2012. Vaccine.

[62] Goldie SJ, O’Shea M, Diaz M, Kim SY (2008). Benefits, cost requirements and cost-effectiveness of the HPV16,18 vaccine for cervical cancer prevention in developing countries: policy implications. Reprod Health Matters.

[63] Levin A, Wang SA, Levin C, Tsu V, Hutubessy R (2014). Costs of introducing and delivering HPV vaccines in Low and lower middle income countries: inputs for GAVI policy on introduction grant support to countries. PLoS One.

[64] Lieu TA, Ray GT, Black SB, Butler JC, Klein JO, Breiman RF (2000). Projected cost-effectiveness of pneumococcal conjugate vaccination of healthy infants and young children. JAMA.

[65] Kim JJ, Sharma M, O’Shea M, Sweet S, Diaz M, Sancho-Garnier H (2013). Model-based impact and cost-effectiveness of cervical cancer preventionin the Extended Middle East and North Africa (EMENA). Vaccine.

[66] Jit M, Brisson M, Portnoy A, Hutubessy R (2014). Cost-effectiveness of female human papillomavirus vaccination in 179 countries: a PRIME modelling study. Lancet Glob Health.

[67] Fesenfeld M, Hutubessy R, Jit M (2013). Cost-effectiveness of human papillomavirus vaccination in low and middle income countries: a systematic review. Vaccine.

[68] Vokó Z, Nagyjánosi L, Kaló Z (2012). Cost-effectiveness of adding vaccination with the AS04-adjuvanted human papillomavirus 16/18 vaccine to cervical cancer screening in Hungary. BMC Public Health.

[69] Kim JJ, Campos NG, O’Shea M, Diaz M, Mutyaba I (2013). Model-based impact and cost-effectiveness of cervical cancer prevention in sub-saharan Africa. Vaccine.

[70] UNICEF At a glance: Panama. Panama first in region to provide free HPV vaccine to young adolescent girls. http://www.unicef.org/infobycountry/panama_46169.html. Accessed 17 Oct 2014.

[71] UNICEF Annual Report 2013 - Sierra Leone. http://www.unicef.org/about/annualreport/files/Sierra_Leone_COAR_2013.pdf. Accessed 17 Oct 2014.

[72] UNICEF Annual Report 2012-Tanzania. http://www.unicef.org/tanzania/TCO_Annual_Report_2012_-_FINAL_June_18_2013.pdf. Accessed 17 Oct 2014.

[73] Zhang SK, Pan XF, Wang SM, Yang CX, Gao XH, Wang ZZ (2013). Perceptions and acceptability of HPV vaccination among parents of young adolescents: A multicenter national survey in China. Vaccine.

[74] Zhao FH, Tiggelaar SM, Hu SY, Zhao N, Hong Y, Niyazi M (2012). A multi-center survey of HPV knowledge and attitudes toward HPV vaccination among women, government officials, and medical personnel in China. Asian Pac J Cancer Prev.

[75] Wang SM, Zhang SK, Pan XF, Ren ZF, Yang CX, Wang ZZ (2014). Human papillomavirus vaccine awareness, acceptability, and decision-making factors among Chinese college students. Asian Pac J Cancer Prev.

[76] Elbasha EH, Dasbach EJ, Insinga RP (2007). Model for assessing human papillomavirus vaccination strategies. Emerg Infect Dis.

[77] Stanley M, Lowy DR, Frazer I (2006). Chapter 12: Prophylactic HPV vaccines: underlying mechanisms. Vaccine.

[78] Gaudet M, Hamm J, Aquino-Parsons C (2014). Incidence of ano-genital and head and neck malignancies in women with a previous diagnosis of cervical intraepithelial neoplasia. Gynecol Oncol.

[79] Sathish N, Wang X, Yuan Y (2014). Human papillomavirus (HPV)-associated oral cancers and treatment strategies. J Dent Res.

[80] Nygård M, Hansen BT, Dillner J, Munk C, Oddsson K, Tryggvadottir L (2014). Targeting human papillomavirus to reduce the burden of cervical, vulvar and vaginal cancerand pre-invasive neoplasia: establishing the baseline for surveillance. PLoS One.

