# Supplementary figures and images for: Estimating long-term clinical effectiveness and cost-effectiveness of HPV 16/18 vaccine in China
Source: BMC Cancer. 2016 Nov 4;16:848. doi: 10.1186/s12885-016-2893-x (PMC5097411; doi:10.1186/s12885-016-2893-x)

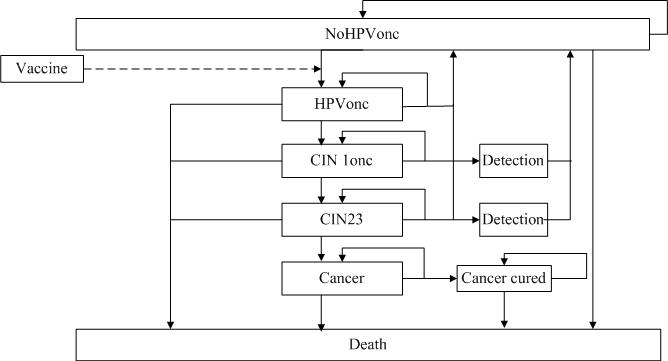

Supplement: Additional file 1: Figure S1. — Lifetime cohort Markov model adapted to the rural and urban settings in China. Note: CIN: cervical intraepithelial neoplasia; CIN1onc: cervical intraepithelial neoplasia 1; HPV: human papillomavirus; HPVonc: oncogenic HPV infection; NoHPVonc: no oncogenic HPV infection. (JPG 24 kb) [file 12885_2016_2893_MOESM1_ESM.jpg]
