# Supplementary material for: Estimating long-term clinical effectiveness and cost-effectiveness of HPV 16/18 vaccine in China
Source: BMC Cancer. 2016 Nov 4;16:848. doi: 10.1186/s12885-016-2893-x (PMC5097411; doi:10.1186/s12885-016-2893-x)
Supplement: Additional file 2: Table S1. — Input data values for base case, one-way sensitivity analyses and probabilistic sensitivity analyses. a Screening practice: all women screened twice lifetime at 35 years and 45 years. Health states No HPV, HPV, CIN 1 and CIN 2/3 have utility=1 (i.e., no disutility); health states death and death from cervical cancer have utility=0; PSA=probabilistic sensitivity analyses; CIN=cervical intraepithelial neoplasia; HPV=human papillomavirus; CC=cervical cancer; VIA/VILI=visual inspection with acetic acid/iodine; NA=Not include in probabilistic sensitivity analyses; Se=sensitivity. (DOCX 21 kb) [file 12885_2016_2893_MOESM2_ESM.docx]

**Table S1. Input data values for base case, one-way sensitivity analyses and probabilistic sensitivity analyses**

| **Parameter** | **Base case value** | | **One-way sensitivity analysis** | | **Distribution and parameters in PSA**  **(SD for normal and range for uniform)** | **References** |
| --- | --- | --- | --- | --- | --- | --- |
|  | **Rural** | **Urban** | **Lower(-20%)** | **Upper(+20%)** |  |  |
| **Transition probabilities** |  |  |  |  |  |  |
| No HPV to HPV | 0-0.19  (Age-specific) | 0-0.11  (Age-specific) | 0-0.16 rural  0-0.09 urban | 0-0.24 rural  0-0.14 urban | Uniform;  (0.00-0.19) in rural;(0.00-0.11) in urban | [43] |
| HPV to No HPV | 0.32-0.61  (Age-specific) | 0.32-0.61  (Age-specific) | 0.26-0.49 | 0.38-0.73 | Uniform; (0.00-0.61) | [43] |
| HPV to CIN1 | 0.05 | 0.05 | 0.05 | 0.05 | Normal; SD=0.01 | [44] |
| CIN1 clearance | 0.50 | 0.50 | 0.50 | 0.50 | Normal; SD=0.15 | [45] [46] |
| CIN1 to CIN2/3 | 0.12 | 0.12 | 0.12 | 0.12 | Normal; SD=0.02 | [45] [46] |
| CIN2/3 clearance | 0.27 | 0.27 | 0.27 | 0.27 | Normal; SD=0.06 | [47] |
| CIN2/3 to cancer | 0.13 | 0.13 | 0.10 | 0.15 | Uniform; (0.10-0.15) | [47] |
| CC death rates (%) | 6.99 | 6.99 | 5.59 | 8.39 | Uniform;(5.59%-8.39%) | [47] [3] |
| Cancer cured | 0.21 | 0.21 | 0.21 | 0.21 | NA | [48] |
| **Screening^a^** |  |  |  |  |  |  |
| Se of VIA/VILI | 37%-55% | - | 30%-44% | 44%-66% | NA | [43] |
| Se of Pap smear | 48%-52% | 48%-52% | 38%-42% | 58%-62% | NA | [52] |
| Se for CIN1 | 44% | 48% | 35% rural  38% urban | 53% rural  58% urban | Uniform;  (35%-53%) in rural;(38%-58%) in urban | [43] [52] |
| Se for CIN2/3 | 52% | 52% | 42% | 62% | Uniform; (42%-62%) | [43] [52] |
| Age at 1st screening | 35 year | 35 year | 30 year | 40 year | NA | Assumption |
| Age at 2nd screening | 45 year | 45 year | 40 year | 50 year | NA | Assumption |
| Screening coverage(%) | 70% | 70% | 56% | 84% | NA | [57][28] |
| CIN1 treated | 0.34 | 0.38 | 0.34 | 0.38 | Uniform;  (0.27-0.40) in rural;(0.31-0.46) in urban | [32] |
| CIN1 cured | 1 | 1 | 1 | 1 | Uniform; (0.8-1.0) | [32] |
| CIN2/3 treated | 0.83 | 0.95 | 0.83 | 0.95 | Uniform;  (0.67-1.00) in rural;(0.76-1.00) in urban | [32] |
| CIN2/3 cured | 0.90 | 0.90 | 0.90 | 0.90 | Uniform; (0.72-1.00) | [32] |
| **Unit costs(CNY)** |  |  |  |  |  |  |
| Screening | 24 | 54 | 19 rural  43 urban | 29 rural  65 urban | Uniform; (19-29) in rural; (43-65) in urban | [32] |
| CIN1 treatment | 367 | 681 | 294 rural  545 urban | 440 rural  817 urban | Uniform;  (294-440) in rural;(545-817) in urban | [32] |
| CIN2/3 treatment | 2,626 | 4,237 | 2,101 rural  3390 urban | 3,151 rural  5084 urban | Uniform;  (2,100-3,151) in rural; (3,390-5,084) in urban | [32] |
| Cancer treatment | 26,715 | 26,715 | 21,372 rural  21,372 urban | 32,058 rural  32,058 urban | Uniform; (21,372-32,058) | [32] |
| Vaccine (3 doses) | 247 | 247 | 87 rural  87 urban | 1,900 rural  1,900 urban | NA | GAVI price, PAHO price, Hong Kong listed price,[33][34] |
| Vaccine administration | 54 | 54 | 43 | 65 | NA | [37] |
| **Disutility scores** |  |  |  |  |  |  |
| CIN1 detected | 0.0128 | 0.0128 | 0.0102 | 0.0154 | Uniform; (0.01-0.02) | [38][39][40][41][42] |
| CIN 23 detected | 0.0128 | 0.0128 | 0.0102 | 0.0154 | Uniform; (0.01-0.02) | [38][39][40][41][42] |
| Cancer treated | 0.273 | 0.273 | 0.218 | 0.328 | Uniform; (0.22-0.33) | [38][39][40][41][42] |
| Cancer cured | 0.062 | 0.062 | 0.050 | 0.074 | Uniform; (0.05-0.07) | [38][39][40][41][42] |
| **Vaccine efficacy (%)** |  |  |  |  |  |  |
| against CC | 93.2 | 93.2 | 78.9 | 98.7 | Normal;SD=0.05 | [49] |
| against CIN2/3 | 64.9 | 64.9 | 52.7 | 74.9 | Normal;SD=0.05 | [49] |
| against CIN1 | 50.3 | 50.3 | 40.2 | 58.8 | Normal;SD=0.05 | [49] |
| **General variables** |  |  |  |  |  |  |
| Discount rate (%) | 3 | 3 | 0 | 5 | NA | [24] |
| Age at vaccination (year) | 12 | 12 | 12 | 18 | NA | Assumption |

^a^Screening practice: all women screened twice lifetime at 35 years and 45 years

Health states No HPV, HPV, CIN 1 and CIN 2/3 have utility=1 (i.e. no disutility); health states death and death from cervical cancer have utility=0

PSA= probabilistic sensitivity analyses; CIN= cervical intraepithelial neoplasia; HPV=human papillomavirus; CC=cervical cancer; VIA/VILI=visual inspection with acetic acid/ iodine; NA=Not include in probabilistic sensitivity analyses; Se=sensitivity
